# Draft Genome Sequence of the Fungus *Penicillium brasilianum* MG11

**DOI:** 10.1128/genomeA.00724-15

**Published:** 2015-09-03

**Authors:** Fabian Horn, Jörg Linde, Derek J. Mattern, Grit Walther, Reinhard Guthke, Axel A. Brakhage, Vito Valiante

**Affiliations:** a Department of Systems Biology/Bioinformatics, Leibniz Institute for Natural Product Research and Infection Biology, Hans Knöll Institute (HKI), Jena, Germany; b Department of Molecular and Applied Microbiology, Leibniz Institute for Natural Product Research and Infection Biology, Hans Knöll Institute (HKI), Jena, Germany; c National Center for Invasive Mycoses, Leibniz Institute for Natural Product Research and Infection Biology, Hans Knöll Institute (HKI), Jena, Germany; d Leibniz Junior Research Group—Biobricks of Microbial Natural Product Syntheses, Leibniz Institute for Natural Product Research and Infection Biology, Hans Knöll Institute (HKI), Jena, Germany; e Friedrich Schiller University, Institute for Microbiology, Jena, Germany.

## Abstract

The genus *Penicillium* belongs to the phylum *Ascomycota* and includes a variety of fungal species important for food and drug production. We report the draft genome sequence of *Penicillium brasilianum* MG11*.* This strain was isolated from soil, and it was reported to produce different secondary metabolites.

## GENOME ANNOUNCEMENT

*Penicillium* is one of the most used genera in biotechnology. It contains well-known species such as *Penicillium chrysogenum*, the major producer of penicillin, and *Penicillium camemberti* and *Penicillium roqueforti*, used in cheese manufacturing (1). *Penicillium brasilianum* is a fungus that grows in soil (2) and on decaying plant material (3, 4). The fungus has been previously reported to produce a wide range of secondary metabolites (5), which show antiviral, cytotoxic, carcinogenic, and insecticidal bioactivity (6, 7). Moreover, the fungus produces a number of different degrading enzymes of biotechnological interest (8).

We sequenced the *P. brasilianum* MG11 strain (7). Phylogenetic analysis of β-tubulin gene sequences, including sequences of ex-type or reference strains of *P. brasilianum*, confirmed the classification of this fungus.

Three different DNA libraries (paired end [PE], 2-kbp mate pair [MP], and 8-kbp MP) of *P. brasilianum* MG11 were sequenced using 2 × 300-bp Illumina MiSeq V3. Reads were adapter clipped, quality trimmed, error corrected (9), and digitally normalized (10). The genome was assembled using the AllPaths-LG assembler (11) and refined using SOAP GapCloser (12) and SEQuel (13). For RNA sequencing, total RNA was extracted from cultures grown in minimal medium for 18 h (shaking cultures) and 72 h (standing cultures) at 30°C. For each sample three biological replicates were sequenced using 2 × 100-bp Illumina HiSeq 2000 (LGC Genomics, Berlin, Germany).

Structural and functional gene prediction followed the protocol previously described (14–16). For the phylogeny-based prediction, the proteomes of *P. bilaiae*, *P. brevicompactum*, *P. chrysogenum*, *P. digitatum*, *P. glabrum*, *P. janthinellum*, *P. lanosocoeruleum*, and *P. raistrickii* (all available at the Joint Genome Institute [JGI]) were mapped to the reference genome. In contrast to other reported studies (15, 16), rRNA was removed from the RNA-Seq data using riboPicker (17). Functional annotations were obtained by using Blast2GO (18) and InterProScan (19) and included the prediction of secondary metabolite gene clusters using SMURF (20). Functional names were suggested by using BLAST and the fungal UniProt Knowledgebase (21).

DNA sequencing of all libraries resulted in 202.6 million reads (15.2 Gbp), from which 130.8 million reads (7.2 Gbp; estimated 205-fold genome coverage) were used for the assembly. RNA sequencing of all libraries resulted in 278.5 million reads (28.0 Gbp), from which 188.2 million reads (18.9 Gbp; estimated 526-fold genome coverage) passed quality filtering and were used for annotation. The assembly procedure resulted in a genome comprising 87 scaffolds (35.9 Mbp; *N*_50_, 3.4 Mbp; *N*_90_, 632.3 kbp). The genome annotation predicted 11,432 genes and 12,343 transcripts. The coding density of the genome is approximately 48%. InterPro domains were assigned to 9,748 transcripts, whereof 3,033 transcripts encode proteins with a predicted transmembrane domain. In 6,173 transcripts at least one gene ontology (GO) annotation was found. GO annotation was made accessible at FungiFun2 (22). Functional annotation suggested names for 4,758 transcripts and assigned 438 transcripts to 36 putative secondary metabolite gene clusters. The annotation contained 477 core eukaryotic genes (23).

### Nucleotide sequence accession numbers.

This genome project was uploaded to DDBJ/ENA/GenBank and is available under the accession numbers CDHK01000001 to CDHK01000087. This paper describes the first version of the genome. Genome data and additional information are also available at the HKI Genome Resource (http://www.genome-resource.de/).
